# Novel Co‐Occurrence of Trisomy 21 and Heterozygous CFTR Mutation

**DOI:** 10.1002/rcr2.70185

**Published:** 2025-04-15

**Authors:** Majd Oweidat, Tamer Qutaina, Alzahra Akram Hamdan, Fatima Zain Hanini

**Affiliations:** ^1^ College of Medicine Hebron University Hebron, West Bank Palestine; ^2^ Department of Pediatrics Al‐Ahli Hospital Hebron, West Bank Palestine

**Keywords:** case report, cystic fibrosis, heterozygous CFTR mutation, trisomy 21

## Abstract

The coexistence of trisomy 21 and cystic fibrosis (CF) is extremely rare, with fewer than 10 reported cases, all involving homozygous CFTR mutations. However, the impact of a heterozygous CFTR mutation in a patient with trisomy 21 remains unexplored. We present a male infant with trisomy 21 who experienced recurrent respiratory distress and was later found to carry a heterozygous pathogenic CFTR mutation (p.Phe508del). His respiratory complications were severe, requiring tracheostomy and long‐term respiratory support. This case highlights the potential interplay between trisomy 21‐associated anatomical features and CFTR‐related airway abnormalities, possibly exacerbating respiratory morbidity. Given the high burden of respiratory complications in both conditions, clinicians should consider CFTR‐related disorders in patients with trisomy 21 presenting with severe respiratory issues. Further research is warranted to determine the clinical significance of CFTR heterozygosity in trisomy 21 and its implications for disease severity and management.

## Graphical abstract

This article reports a novel co‐occurrence of trisomy 21 and a heterozygous CFTR mutation in an infant.

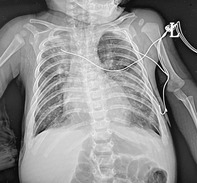

## 1 Introduction

Cystic fibrosis (CF) is a common inherited autosomal recessive disorder, affecting 0.347 per 10,000 people in North America. It results from a defective CF transmembrane conductance regulator (CFTR) protein, caused by mutations in the CFTR gene located on the long arm of chromosome 7 [1, 2].

Down syndrome, also known as trisomy 21, is the most frequent autosomal genetic disorder affecting about 1 per 800 live babies [3]. The chance of having a baby with trisomy 21 increases as the mother gets older [4]. Most people with trisomy 21 have all three copies of chromosome 21, and this happens when chromosomes do not separate correctly during cell division. Some less common types of trisomy 21 are translocation and mosaic trisomy 21 [5]. People with trisomy 21 can have various physical features and health conditions. Some may experience heart issues, such as atrioventricular septal defects, or blood cancers. In addition to these concerns, individuals with trisomy 21 are particularly susceptible to complex and recurrent respiratory problems, with respiratory symptoms occurring more frequently compared to the general population (30% vs. 15.2%) [6, 7].

Herein, we report a novel co‐occurrence of trisomy 21 and a heterozygous CFTR mutation in an infant.

## 2 Case Report

A male infant with trisomy 21, diagnosed based on clinical features and later confirmed as full trisomy 21 by chromosomal analysis, presented to our paediatric unit at 5 months of age with recurrent respiratory issues. The patient's clinical features included upward‐slanting palpebral fissures, epicanthal folds, a flat nasal bridge, low‐set round ears, a short neck with excess nuchal skin, and a single transverse palmar crease. His prenatal history was notable for being delivered at 36 weeks of gestation by normal spontaneous vaginal delivery after an uncomplicated pregnancy to a 44‐year‐old mother, gravida 10, para 9, abortus 1 (G10P9A1). Nuchal translucency measurement at 12 weeks of gestation showed no concerning findings.

At birth, the patient cried immediately, passed urine and meconium within the first 24 h, and had an Apgar score of 7 and 9 at 1 and 5 min, respectively. His birth weight was 3085 g, and he showed cyanosis shortly after birth. He was admitted to a neonatal intensive care unit for 3 days due to suspected sepsis and congenital heart disease. An echocardiogram confirmed a complete balanced atrioventricular canal defect that underwent surgical repair later and a small patent ductus arteriosus. He was placed on regular follow‐up with a cardiologist and maintained on *Furosemide* and *Captopril* for heart failure management.

At 1 month old, during his second admission, the patient was experiencing several respiratory infections and was managed with hypertonic saline nebulization, nasal sprays, and antibiotics, with partial symptom resolution. Notably, his family history included a sister diagnosed with CF, homozygous for the common p.Phe508del mutation. This history gained significance as the case progressed, particularly as suspicion of a CF‐related disorder was raised, despite the initial presentation being more consistent with his underlying trisomy 21 and congenital heart disease. Additionally, the sister had recently passed away at the age of 23.

**FIGURE 1:**
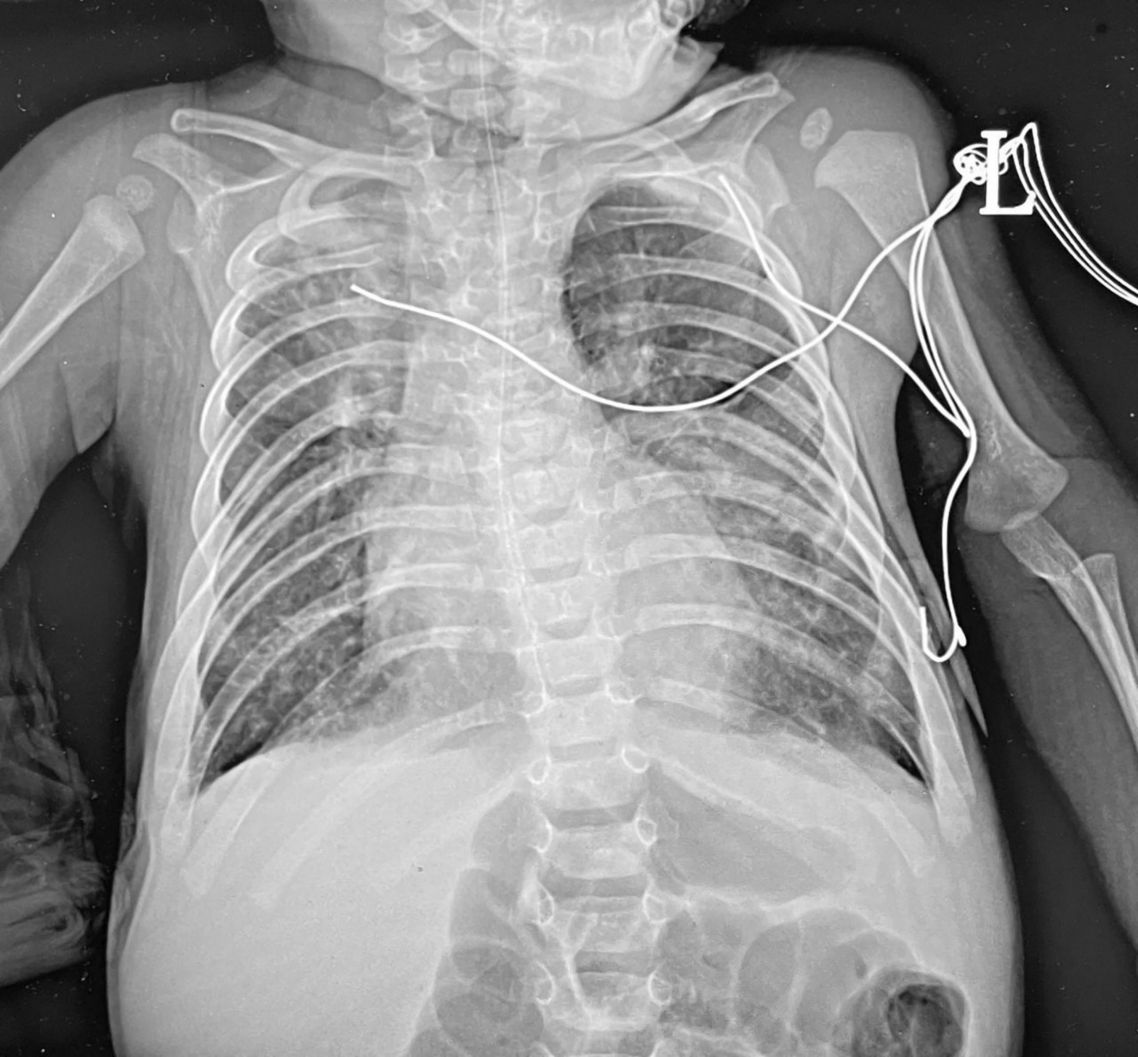
A chest x‐ray shows bilateral diffuse infiltrates consistent with chronic lung disease, along with scattered opacities throughout both lungs. Additionally, there is right upper lobe atelectasis and a mild right‐sided pleural effusion, characterised by blunting of the costophrenic angle.

In the third and most severe presentation, the patient was referred to our unit with worsening cough, noisy breathing, and cyanosis over the course of 2 days. The family reported 4 days of fever, poor feeding, and lethargy. On arrival, the infant showed respiratory distress with subcostal retractions, right‐sided decreased air entry, and bilateral wheezes and crackles. Initial workup revealed right upper lobe infiltration on chest x‐ray, as shown in Figure 1, and he was admitted with the diagnosis of suspected chest infection for further management. His hospital course was complicated by persistent respiratory distress, recurrent episodes of desaturation, and failure to extubate despite multiple attempts. On day 26 of admission, he underwent a tracheostomy and gastrostomy for long‐term respiratory support and feeding. Post‐tracheostomy chest x‐ray showed increased lung vascularity with no signs of acute lung pathology.

Sweat test was done and resulted in elevated sweat chloride levels (65 mmol/L), confirming the diagnosis of CF. Concurrently, a genetic test for CF was initiated. The patient developed acute kidney injury during his hospital stay, managed with fluid restriction and dose adjustments of diuretics. He also experienced recurrent infections with leukocytosis, requiring multiple courses of antibiotics, including *Vancomycin*, *Piperacillin‐Tazobactam*, and *Azithromycin*. His haemodynamic instability necessitated vasopressor support, with gradual improvement and stabilisation by the time of discharge. During his hospital stay, he was also found to have hyperbilirubinemia, which was managed with *Ursodeoxycholic acid (UDCA)* and vitamin supplementation.

A major turning point occurred when the CFTR gene Whole Exome Sequencing (WES) results revealed the patient to be heterozygous (carrier) for the common pathogenic variant CFTR (NM_000492.4):c.1521_1523del(p.Phe508del)). This raised the possibility of a CFTR‐related disorder, particularly in light of his respiratory complications. His ongoing respiratory issues were managed with long‐term supplemental oxygen through the tracheostomy, nebulized bronchodilators, and *Azithromycin* prophylaxis. Full feeding was achieved via gastrostomy, supplemented with medium‐chain triglycerides oil and vitamins. Pancreatic function testing did not indicate pancreatic insufficiency.

At discharge, the patient was hemodynamically stable, with normal saturation levels on 2 L of oxygen via tracheostomy. He remained on *Sildenafil* for pulmonary hypertension, alongside diuretics and nutritional support. A follow‐up plan was established for respiratory and cardiac management, and genetic counselling was recommended for the family due to the CF carrier status and risk for future pregnancies.

## 3 Discussion

This article presents a 5‐month‐old male with full trisomy 21 who presented with recurrent respiratory distress. Despite typical Down syndrome features, elevated sweat chloride and a heterozygous CFTR mutation were identified.

The most prevalent mutation associated with CF is ΔF508. It accounts for approximately 70% of all CFTR mutations and is present in about 85% of CF patients worldwide [1, 2]. Initial symptoms of CF may include meconium ileus, failure to thrive, and signs of malabsorption. As individuals grow older, they often face recurrent respiratory infections, episodes of pancreatitis, and infertility [8].

Heterozygous CFTR and trisomy 21 mutations each contribute to respiratory complications through distinct yet potentially synergistic mechanisms. Heterozygous CFTR mutations, while not causing classical CF, exacerbate respiratory dysfunction by impairing the CFTR protein's role in maintaining airway hydration and mucociliary clearance [9]. This creates an environment prone to recurrent infections, inflammation, and chronic respiratory conditions such as bronchiectasis and allergic bronchopulmonary aspergillosis [9]. Carriers of CFTR mutations also experience increased rates of respiratory infections and hospitalisations, with potential risks of antibiotic resistance due to frequent infections [9].

Trisomy 21 predisposes individuals to respiratory issues due to anatomical anomalies and immune dysregulation. Structural abnormalities such as a narrower trachea, hypoplastic lungs with reduced alveoli and branching, and upper airway issues like macroglossia and adenotonsillar hypertrophy lead to airway obstruction, recurrent infections, and conditions like obstructive sleep apnea (OSA) [10]. Immune dysfunction, driven by hyperactive interferon (IFN) signalling from overexpression of IFN receptors on chromosome 21, results in a pro‐inflammatory state, poor viral clearance, and heightened susceptibility to severe infections such as RSV [10]. Together, these genetic conditions may have additive or synergistic effects, compounding airway obstruction and immune challenges.

To the best of our knowledge, the co‐occurrence of trisomy 21 and CF has been documented in fewer than 10 patients; however, none of these cases involved heterozygous individuals [11]. This represents the first globally reported instance of a patient with a heterozygous CF mutation co‐occurring with trisomy 21, which may influence CF severity and clinical course.

The variant reported in our case is classified as pathogenic by the American College of Medical Genetics and Genomics (ACMG) as mentioned on the ClinVar platform. While the patient showed almost typical features of both conditions, the heterozygous CFTR mutation could potentially result in a milder form of CF [9]. However, its exact role in the context of trisomy 21 remains speculative without functional studies or additional case data. We think that the presence of trisomy 21 may exacerbate CF‐related complications, presenting a unique clinical challenge. Further research is needed to clarify the interaction between these genetic factors and their impact on clinical outcomes.

Emerging therapies like *vanzacaftor‐tezacaftor‐deutivacaftor* have shown promise in managing CFTR‐related dysfunction [12]. Although they have primarily been studied in homozygous or compound heterozygous F508del‐CFTR mutations.

The prognosis for individuals with both trisomy 21 and CF has been historically very poor. Most affected children have died during infancy, with the oldest reported survivor reaching 6 years and 11 months [11].

This case highlights the novel co‐occurrence of trisomy 21 and a heterozygous CFTR mutation, emphasising the potential for compounded respiratory morbidity due to synergistic anatomical and CFTR‐related dysfunction. It highlights the importance of considering CFTR‐related disorders in trisomy 21 patients with severe respiratory issues. This report adds to the limited literature by exploring the clinical implications of CFTR heterozygosity in trisomy 21, advocating for further research to guide management and improve outcomes.

## Author Contributions

**Majd Oweidat:** principal investigator, conceived and designed the study, interpreted the data, and wrote, drafted, and revised the manuscript. **Tamer Qutaina:** served as the senior researcher and supervised all the work steps. **Alzahra Akram Hamdan:** contributed to data collection and literature review. **Fatima Zain Hanini:** contributed to data collection and literature review.

## Ethics Statement

The authors declare that appropriate written informed consent was obtained for the publication of this manuscript and accompanying images.

## Conflicts of Interest

The authors declare no conflicts of interest.

## Data Availability

Data sharing is not applicable to this article as no new data were created or analyzed in this study.
